# Supplementary material for: Microbiota-derived acetate enhances host antiviral response via NLRP3
Source: Nat Commun. 2023 Feb 6;14:642. doi: 10.1038/s41467-023-36323-4 (PMC9901394; doi:10.1038/s41467-023-36323-4)
Supplement: Supplementary file 1 — Supplementary Information [file 41467_2023_36323_MOESM1_ESM.pdf]

*Supplementary Information*

**Microbiota-derived acetate enhances host antiviral response via NLRP3**

Junling Niu[1,2], Mengmeng Cui[1], Xin Yang[2], Juan Li[1,3], Yuhui Yao[1,4], Qiuhong Guo[1], Ailing Lu[1], Xiaopeng Qi[5], Dongming Zhou[1], Chenhong Zhang[2,*], Liping Zhao[2,6,*], Guangxun Meng[1,3,4,*]

*Corresponding authors.

E-mail: gxmeng@ips.ac.cn (G. Meng); liping.zhao@rutgers.edu (L. Zhao); zhangchenhong@sjtu.edu.cn (C. Zhang)

## Supplementary Figures and Figure legends

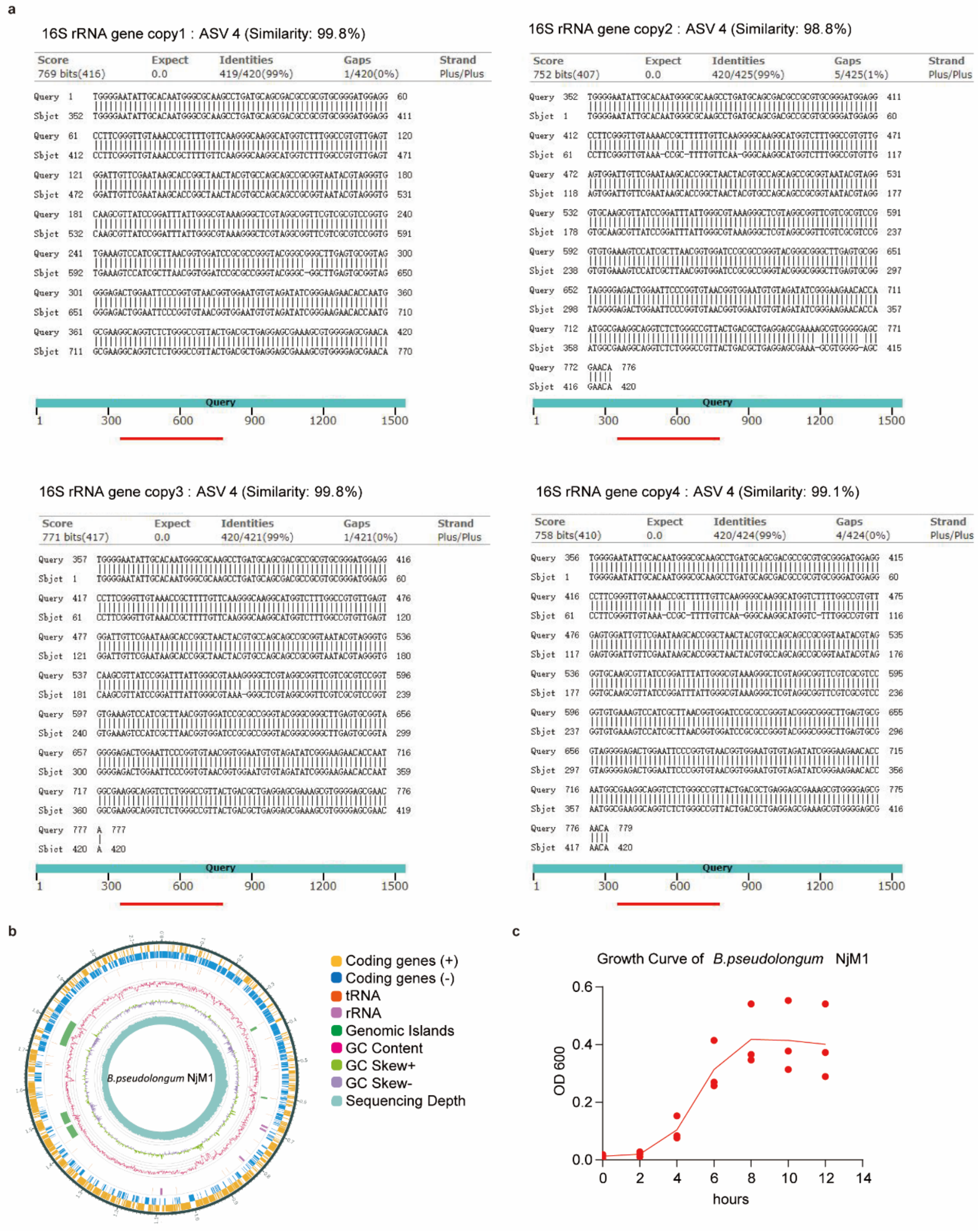


**Supplementary Fig. 1 Identification of *B. pseudolongum* NjM1. a,** Alignment results of 16S rRNA sequences in the genome of *B. pseudolongum* NjM1 strain with ASV4. **b,** Genome atlas of *B. pseudolongum* NjM1. From outer to inner: coding genes (+, yellow), coding genes (-, blue), tRNAs (orange),

rRNAs (purple), genomic islands (green), GC content (purple-red), GC-skew and sequencing depth (light blue). **c,** The growth curve of *B. pseudolongum* NjM1 was determined by measuring OD600. Results represent n=3 independent experiments (**c**). Source data are provided as a Source Data file.

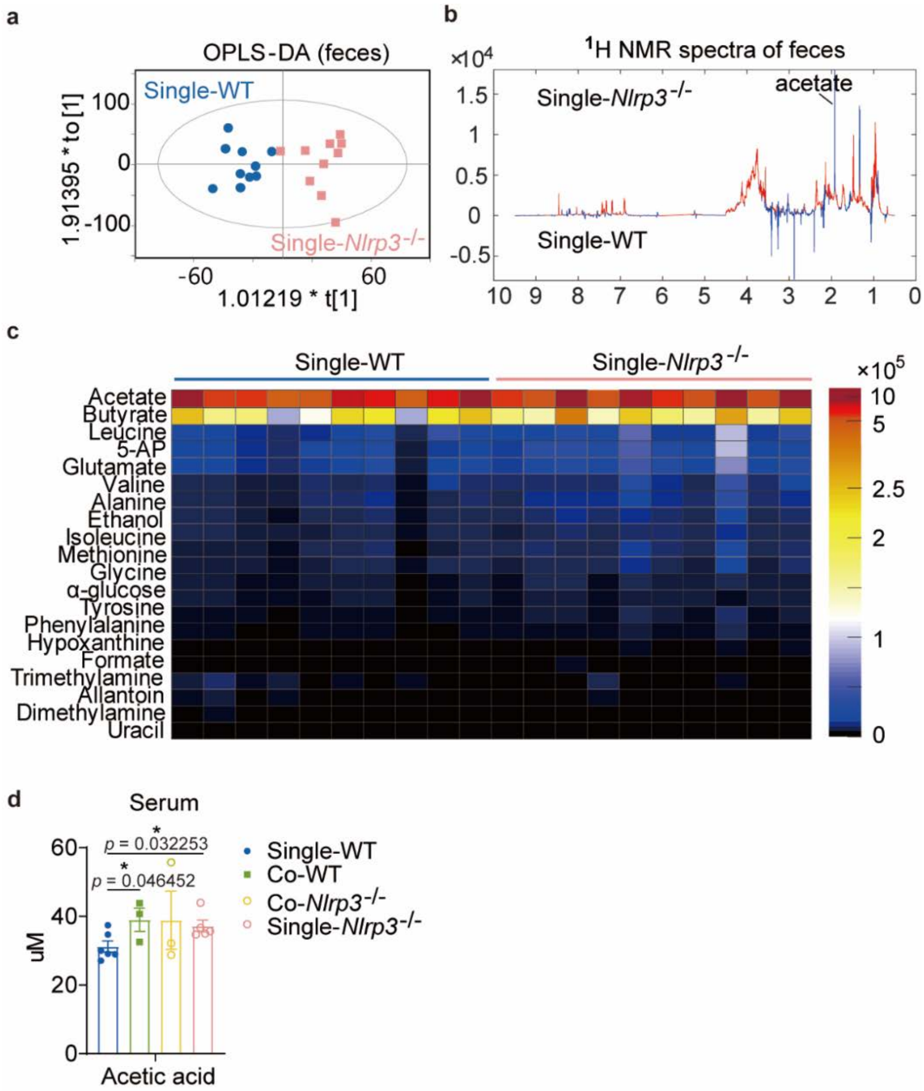


**Supplementary Fig. 2 Fecal and serum concentration of acetate in Single-*Nlrp3*$^{-/-}$ mice is higher than in Single-WT. a-c,** Cross-validated OPLS-DA scores plots (**a**) and the corresponding loadings plots (**b**) and heat maps (**c**) of feces extracts data from 9-week-old Single-WT mice and Single-*Nlrp3*$^{-/-}$ mice. (n=10 per group, OPLS-DA: $Q^2$ = 0.563. CV-ANOVA: P =0.010). 5-AP, aminopentanoate. **d,** *Nlrp3*$^{-/-}$ (n=6 or 3) and WT (n=6 or 3) mice were either singly housed or co-housed at a 1:1 ratio from weaning to 9 weeks of age. Quantitative detection of acetate in peripheral blood by GC-FID/MS was conducted by Shanghai Metabolome Institute (SMI)-Wuhan using an optimized method previously reported (Furuhashi et al., *Anal. Biochem.* 543, 51-54, 2018).

Results represent n=2 independent experiments (**d**). Data in **d** are presented as mean ± SEM, one-way ANOVA with Dunnett’s post-hoc test. Significant values are defined by *$p < 0.05$. Source data are provided as a Source Data file.

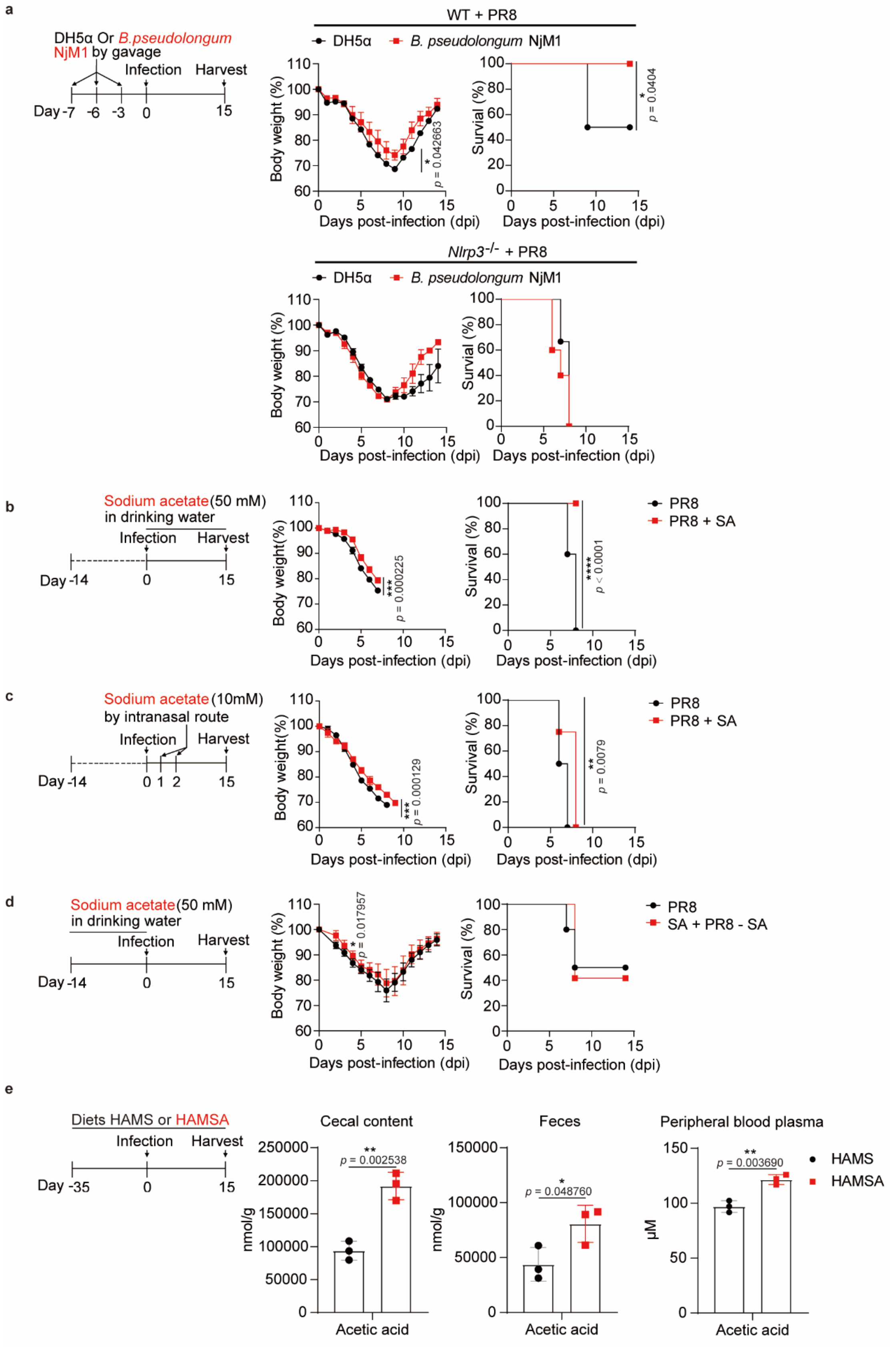


**Supplementary Fig. 3 Acetate protects against IAV infection in various applications. a,** WT (n=6, n=6) and *Nlrp3*$^{-/-}$ mice (n=10, n=6) were given either

$1.48 \times 10^9$ *B. pseudolongum* NjM1 or DH5α for each mouse by gavage 3 times, and then intranasally infected with influenza A virus PR8 (H1N1). **b-c,** WT mice were given either 50 mM sodium acetate (n=10) or drinking water (n=10) and simultaneously infected with IAV (**b**) or inoculated with 10 mM sodium acetate (n=8) or drinking water (n=8) post-intranasal IAV instillation (**c**). **d,** WT mice were treated with sodium acetate that was added to the drinking water before infection and withdrawn after infection (n=8) or with drinking water as control during infection (n=10). Body weight changes in percentage and survival rates of such mice post infection were assessed (**a-d**). **e,** WT mice were given diets HAMS (n=8) or HAMSA (n=8) for 5 weeks and then intranasally infected with influenza A virus PR8 (H1N1). Cecal, fecal samples and blood were processed for SCFA analysis using gas chromatography at the end of the viral infection. Results represent n=2 independent experiments (**a-e**). Changes in body weights in **a-d** and acetic acid concentrations in **e** are presented as mean ± SEM, two-tailed Student's *t* test. Survival rates shown in **a-d** are analyzed with Log-rank (Mantel-Cox) test. Significant values are defined by $*p < 0.05$, $** p < 0.01$, $*** p < 0.001$, $**** p < 0.0001$. Source data are provided as a Source Data file.

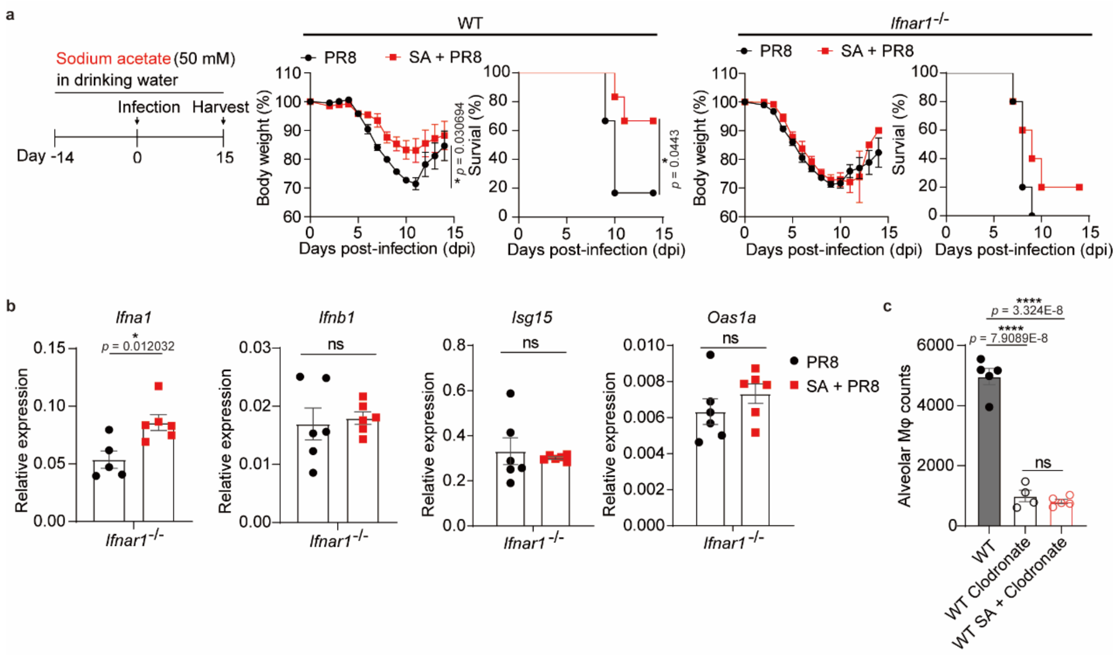


**Supplementary Fig. 4 Acetate suppresses IAV replication in an Ifnar1-dependent manner. a,** WT and *Ifnar1*$^{-/-}$ mice were given drinking water with or without 50 mM sodium acetate (WT, n=6 or 6; *Ifnar1*$^{-/-}$, n=5 or 5) and then intranasally infected with influenza A virus PR8 (H1N1). Body weight changes in percentage and survival rates of such mice post infection were assessed. This is a different repeat from Fig.4a. **b,** The relative expression of *Ifna1*, *Ifnb1, Ifng, Isg15* and *Oas1a* to *Gapdh* in the lung homogenates from infected *Ifnar1*$^{-/-}$ mice was determined by qPCR on day 7 post infection (*Ifna1* n=5, n=6; *Ifnb1* n=6, n=6; *Isg15* n=6, n=6; *Oas1a* n=6, n=6). **c,** Flow cytometry analysis of alveolar macrophage counts of clodronate-treated (n=4, n=5) or untreated WT (n=5) mice on day 3 post infection. Results represent n=3 independent (**a-b**) and n=2 independent experiments (**c**). Changes in body weights in **a** are presented as mean ± SEM, two-tailed Student's *t* test. Survival rates are analyzed with Log-rank (Mantel-Cox) test. Data in **b-c** are presented as mean ± SEM, two-tailed Student's *t* test (**b**), one-way ANOVA with Tukey's post-hoc test (**c**). Significant values are defined by *$p < 0.05$, ** $p < 0.01$, *** $p < 0.001$,

**** $p < 0.0001$. ns, not significant. Source data are provided as a Source Data file.

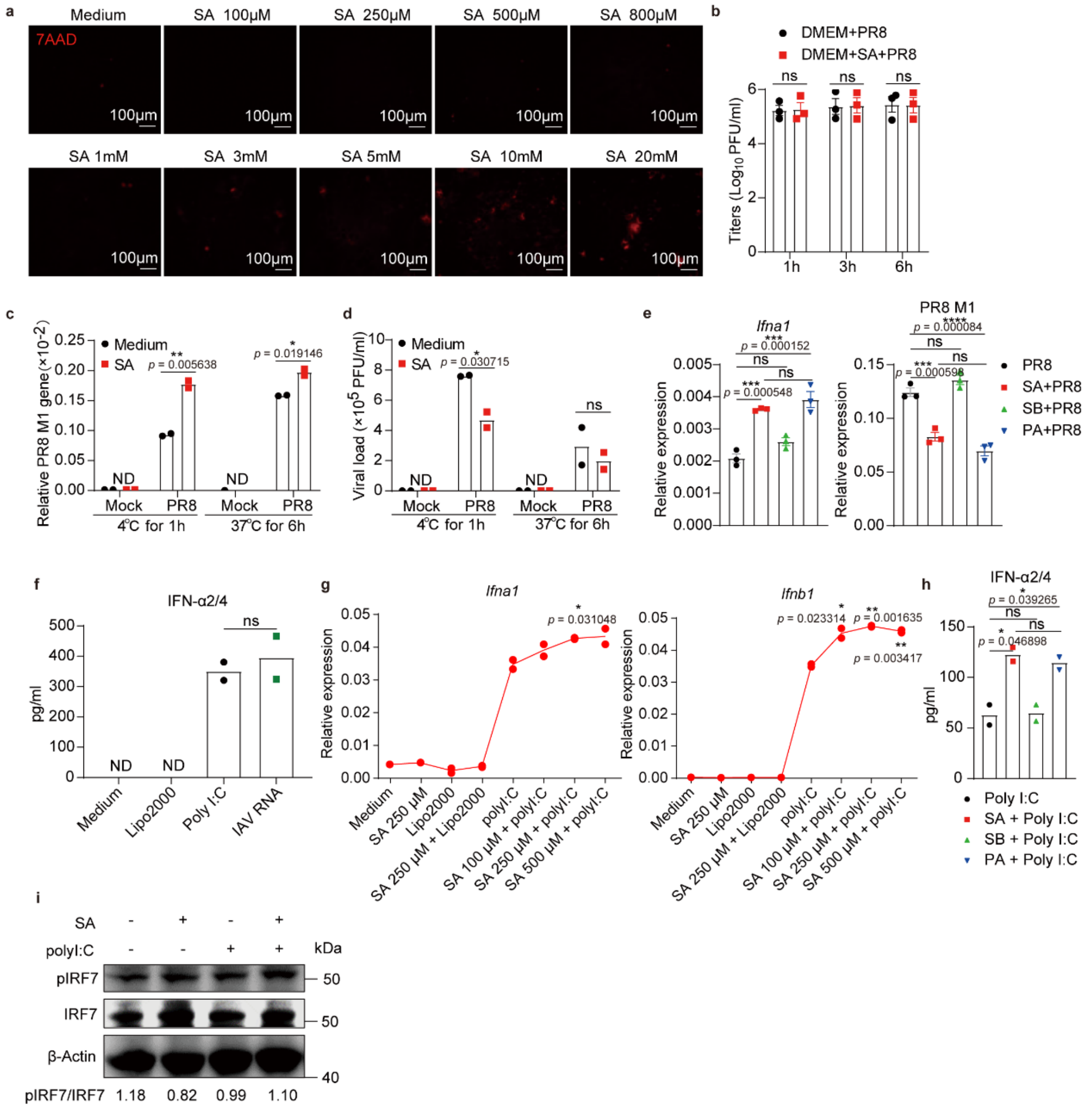


**Supplementary Fig. 5 Acetate enhances the production of IFN-I and containment of influenza virus in macrophages. a,** BMDMs were treated with SA for 72 h and stained with 7AAD. Fluorescence images were captured for an indication of cell death. **b,** PR8 particles were incubated with 250 μM SA for 1 h, 3 h or 6 h, and then infected MDCK after 10-fold dilution for 48 h. Viral titer was determined with the standard plaque assay (n=3). **c-d,** BMDMs were pretreated with 250 μM SA for 24 h at 37℃ and then infected with PR8 (MOI=2) for 1 h at 4℃ or for 6 h at 37℃: (**c**) Relative PR8 *M1* gene expression to *Gapdh* was determined by qPCR (n=2); (**d**) Viral load in the supernatant of infected

BMDMs was determined with the standard plaque assay (n=2). **e, h,** BMDMs were pretreated with 250 μM sodium acetate (SA), sodium butyrate (SB), potassium acetate (PA) for 24 h and then infected with PR8 (MOI=2) for 24 h (**e**) or transfected with 200 ng polyI:C for 12 h (**h**). Relative *Ifna1* and PR8 *M1* gene expression to *Gapdh* (**e**) (n=3) and concentrations of IFN-α2/4 (**h**) (n=2) were determined. **f,** BMDMs were transfected with 200 ng polyI:C or 100 ng IAV RNA using Lipofectamine 2000 reagent for 12 h. IFN-α2/4 released from BMDMs was determined by ELISA (n=2). **g, i,** BMDMs were pretreated with SA for 24 h and then transfected with 200 ng polyI:C for 6 h (**g**) or 1 h (**i**). Relative *Ifna1* and *Ifnb1* expression to *Gapdh* was determined (**g**) (n=2). The cell lysates were subjected to immunoblot analysis and the relative ratios of pIRF7 (Ser477) to total IRF7 were marked below (**i**). Results represent n=3 independent (**a-d, f-g**) and n=2 independent experiments (**e, h, i**). Data in **b-h** are presented as mean (± SEM), two-tailed Student's *t* test (**b-d, f, g**). one-way ANOVA with Tukey's post-hoc test (**e, h**). Significant values are defined by $^{*}p < 0.05$, ** $p < 0.01$, *** $p < 0.001$, **** $p < 0.0001$. Source data are provided as a Source Data file.

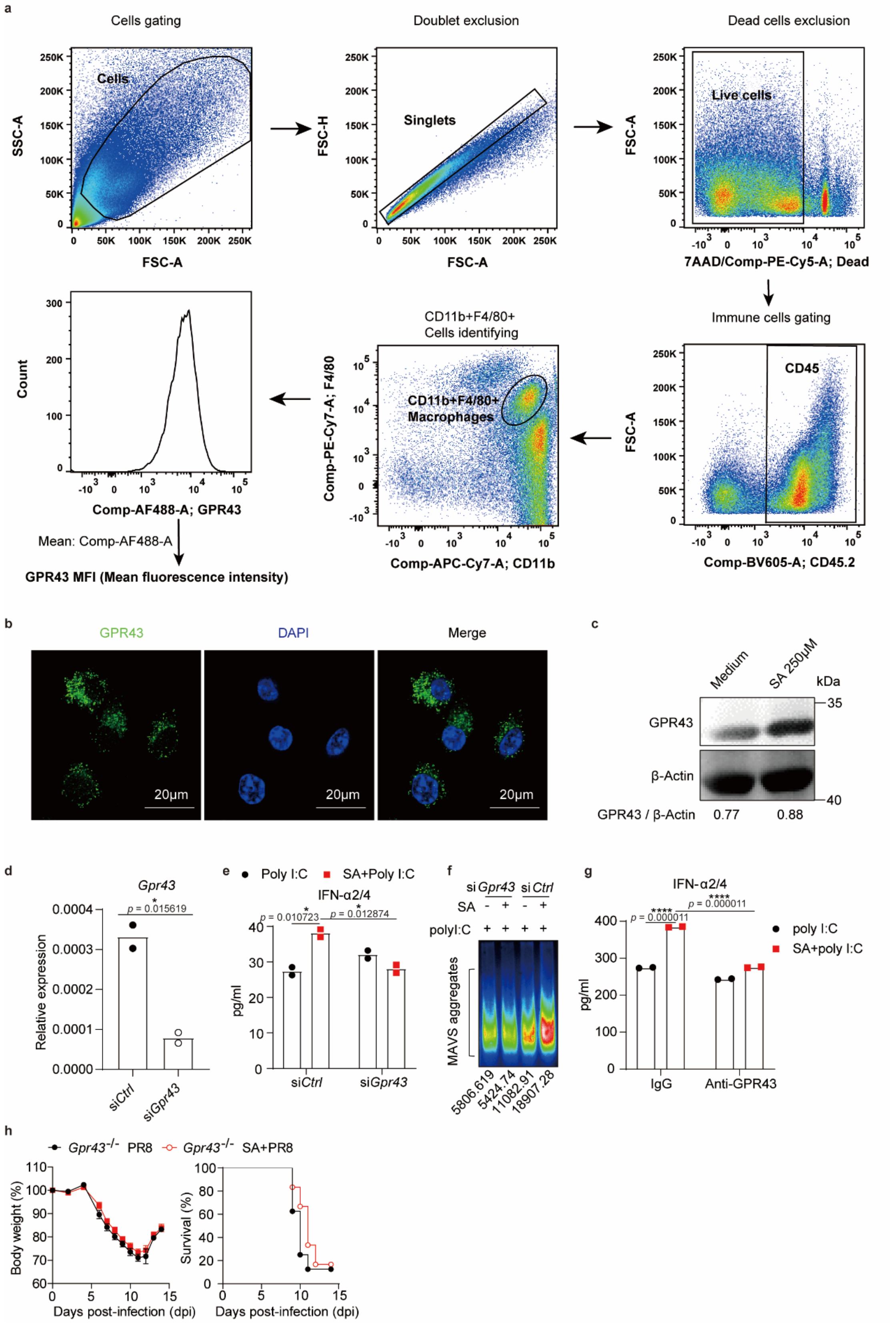


**Supplementary Fig. 6 Acetate promotes IFN-I production through GPR43.**

**a,** Gating strategy used in flow cytometry analysis to detect CD11b$^{+}$F4/80$^{+}$

macrophages in BALF. Cell debris were excluded by SSC-A and FSC-A gating (Cells), doublets were excluded by FSC-A and FSC-H gating (Singlets), dead cells were excluded by FSC-A and 7AAD gating (Live cells), live cells were gated on leukocyte population (CD45+), and the subset of macrophages was estimated according to the markers CD11b and F4/80. Mean fluorescence intensity of GPR43 on CD11b+F4/80+ macrophages was calculated by adding statistic Mean of Comp-AF488-A, corresponding to Figure 6c. **b,** Macrophages were differentiated from bone marrow of 6- to 8-week-old WT mice, fixed with 4% paraformaldehyde, blocked with 1% BSA and then stained with AF488-conjugated rabbit anti-GPR43, and fluorescence images of GPR43 (green) and cell nuclei (blue) were captured with confocal microscope. **c,** BMDMs were pretreated with 250 μM SA for 24 h and cell lysates were subjected to immunoblot analysis with indicated antibodies. The ratios of GPR43 to β-Actin were marked below. **d-e,** $1\times10^6$ BMDMs were transfected by electroporation with 100 pmol small interfering RNA targeting *Gpr43* (si*Gpr43*) or *Luciferase GL2* (si*Ctrl*). **d,** After 48 h, *Gpr43* mRNA was determined (n=2). **e,** After 48 h, the BMDMs were treated, stimulated and analyzed as in Fig. **5e** (n=2). **f,** BMDMs were transfected, treated and stimulated as in **e**. After stimulation with polyI:C for 1 h, crude mitochondria extracts were subjected to SDD–AGE as in Fig. **5h**. **g,** BMDMs were treated with rabbit anti-GPR43 (200 ng/mL) or rabbit polycolonal IgG (200 ng/mL) for 1 h, and then treated, stimulated and analyzed as in Fig. **5e** (n=2). **h,** *Gpr43*$^{-/-}$ mice were given drinking water with or without 50 mM SA (n=6 or 8), and then intranasally infected with PR8. Body weight changes in percentage and survival rates of such mice post infection were assessed. Results represent n=2 independent experiments (**b-h**). Data in **d, e,**

**g** and changes in body weights shown in **h** are presented as mean ± SEM, two-tailed Student's *t* test (**d, h**), one-way ANOVA with Dunnett's post-hoc test (**e, g**). Survival rates shown in **h** are analyzed with Log-rank (Mantel-Cox) test. Significant values are defined by *$p < 0.05$, ** $p < 0.01$, *** $p < 0.001$, **** $p < 0.0001$. Source data are provided as a Source Data file.

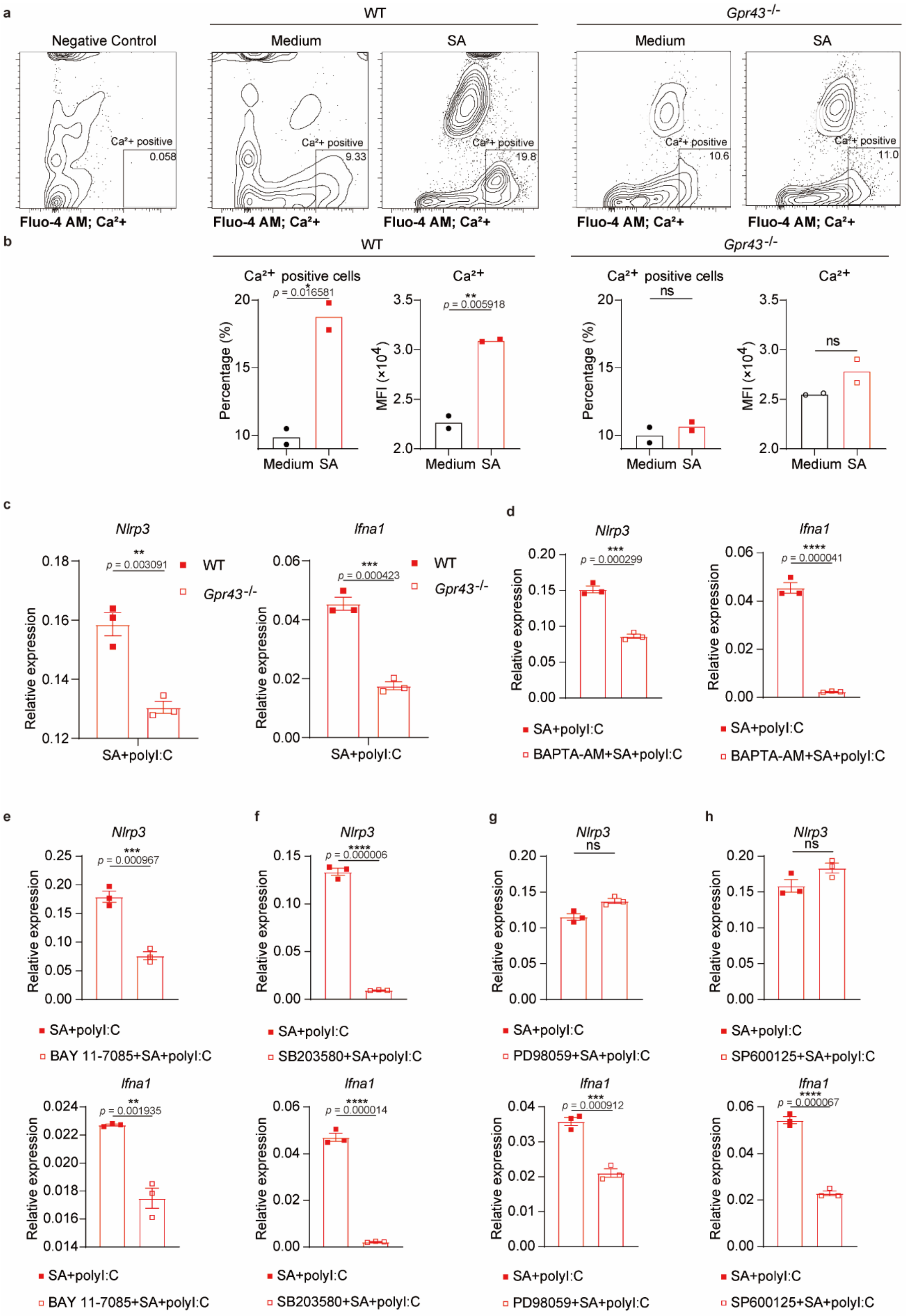

**Supplementary Fig. 7 Acetate upregulates Nlrp3 expression through GPR43 activation. a-b,** WT or *Gpr43*$^{-/-}$ BMDMs were treated with 250 μM sodium acetate (SA) or medium for 24 h, washed with PBS three times, and

then incubated with 2 μM of Fluo-4 AM (a $Ca^2$+ fluorescence probe) diluted in PBS for 45 min at 24℃. The percentage of $Ca^2$+ positive cells (**a, b**) and mean fluorescence intensity (MFI) of $Ca^2$+ (**b**) was analyzed by flowcytometry. **c,** WT and *Gpr43*$^{-/-}$ BMDMs were pretreated with 250 μM SA for 24 h and then transfected with 200 ng polyI:C. Relative *Nlrp3* and *Ifna1* expression to *Gapdh* was determined by quantitative real-time PCR (n=3). **d-h,** WT BMDMs were pretreated with 10 μM BAPTA-AM (a $Ca^2$+ chelator), 10 μM BAY 11-7085 (a NF-κB inhibitor), 10 μM SB203580 (a MAPK P38 inhibitor), 20 μM PD98059 (a MAPK ERK inhibitor), or 25 μM SP600125 (a MAPK JNK inhibitor) for 1 h and 250 μM SA for 24 h and then transfected with 200 ng polyI:C. Relative *Nlrp3* and *Ifna1* expression to *Gapdh* was determined by quantitative real-time PCR (n=3). Results represent n=2 independent experiments (**a-h**). Data in **b-h** are presented as mean ± SEM, two-tailed Student's *t* test. Significant values are defined by *$p < 0.05$, ** $p < 0.01$, *** $p < 0.001$, **** $p < 0.0001$. ns, not significant. Source data are provided as a Source Data file.

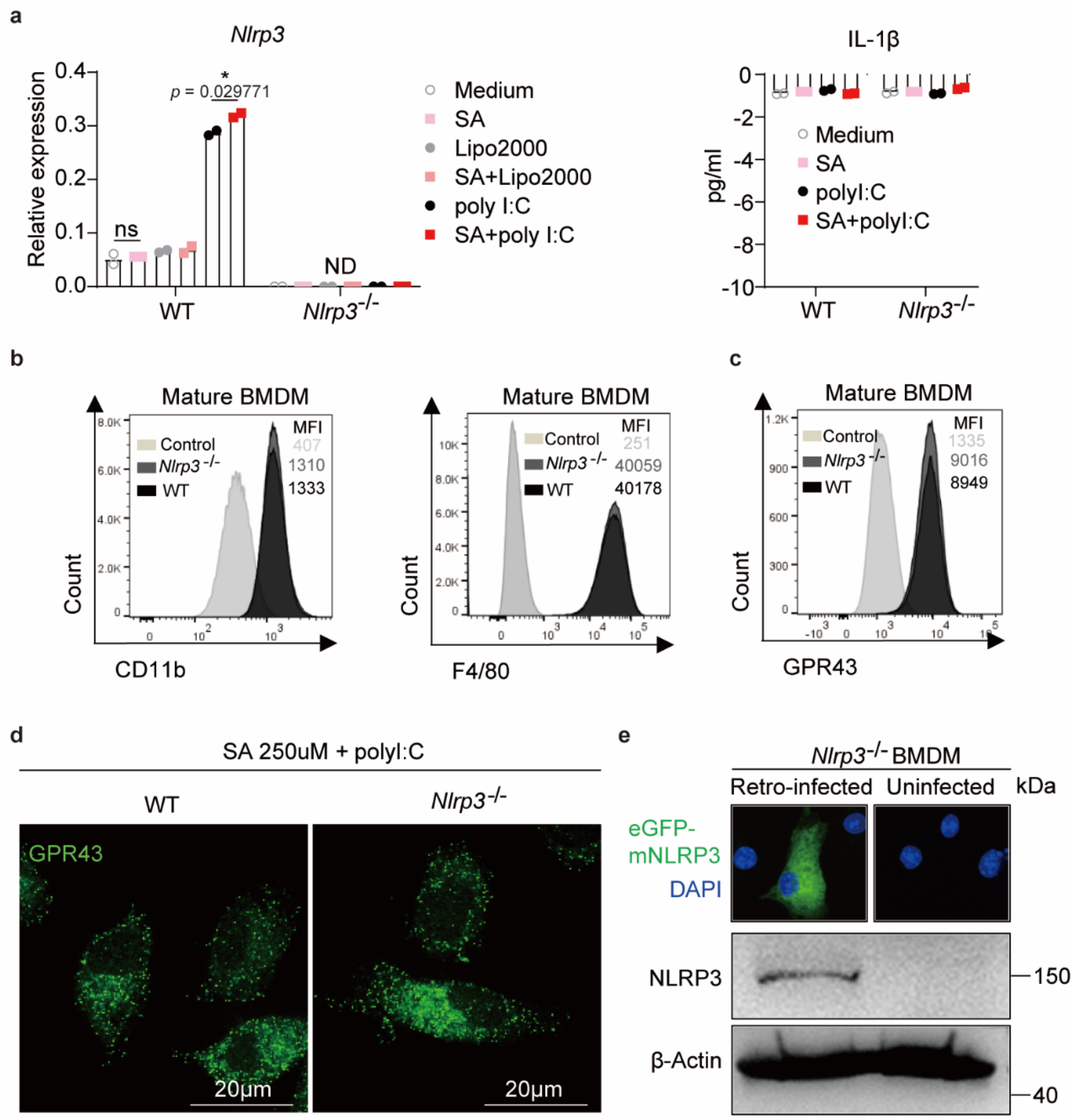


**Supplementary Fig. 8 *Nlrp3* deficiency has no effect on the expression of surface markers or GPR43 on macrophages and Retrovirus-mediated eGFP-mNlrp3 expression were confirmed in *Nlrp3*$^{-/-}$ BMDM. a,** WT and *Nlrp3*$^{-/-}$ BMDMs were pretreated with or without 250 μM sodium acetate for 24 h and then transfected with 200 ng polyI:C for 12 h. *Nlrp3* mRNA expression was determined by qPCR and used to confirm *Nlrp3* deficiency (n=2). Concentrations of IL-1β released from BMDMs were determined by ELISA (n=2). ND, not detected. **b-c,** Macrophages were differentiated from bone marrow of 6- to 8-week-old WT or *Nlrp3*$^{-/-}$ mice with 30% L929 conditioned

medium and stained with indicated antibodies shown in Flow Cytometry section and analyzed on a flow cytometer. **d,** WT and *Nlrp3*$^{-/-}$ BMDMs were pretreated with 250 μM sodium acetate for 24 h and then transfected with polyI:C for 1 h. The BMDMs were fixed with 4% paraformaldehyde (PFA), blocked with 1% BSA and then stained with AF488-conjugated rabbit anti-GPR43. Fluorescence images of GPR43 (green) were captured with confocal microscope. **e,** *Nlrp3*$^{-/-}$ BMDM were infected with PRP-eGFP-mNlrp3 retrovirus, eGFP-mNlrp3 expression were confirmed. Results represent n=3 independent (**a**) and n=2 independent experiments (**b-e**). Data in **a** are presented as mean ± SEM, two-tailed Student's *t* test. Significant values are defined by $*p < 0.05$. Source data are provided as a Source Data file.

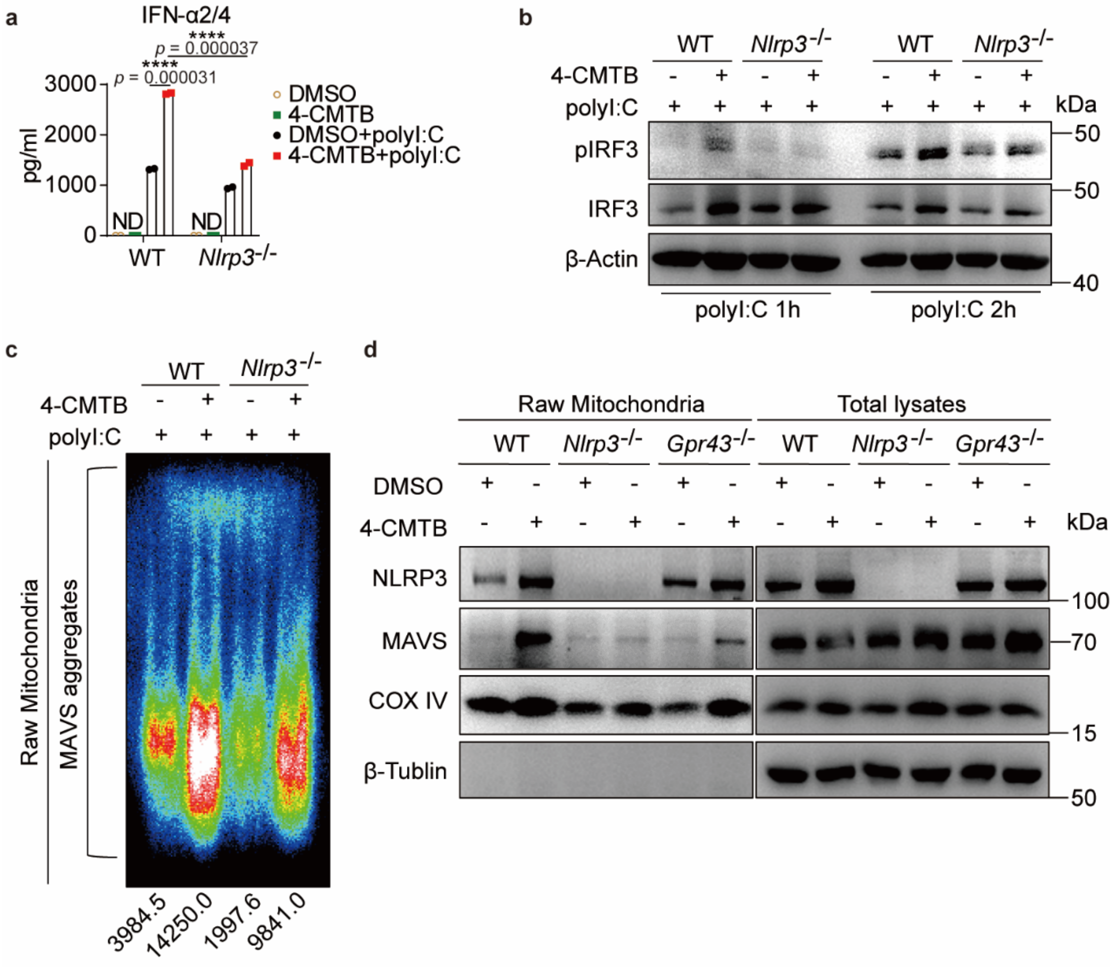


**Supplementary Fig. 9 NLRP3 mediates translocation of MAVS triggered by GPR43 activation to mitochondria. a,** WT and *Nlrp3*$^{-/-}$ BMDMs were pretreated with or without 20 μM 4-CMTB for 24 h, and then transfected with 200 ng polyI:C for 12 h. Concentrations of IFN-α2/4 released from BMDMs were determined by ELISA (n=2). one-way ANOVA with Dunnett's post-hoc test. Significant values are defined by **** $p < 0.0001$. **b,** WT or *Nlrp3*$^{-/-}$ BMDMs were pretreated with or without 20 μM 4-CMTB for 24 h and then transfected with 200 ng polyI:C for 1 h or 2 h. The cell lysates were subjected to immunoblot analysis with indicated antibodies. **c,** WT or *Nlrp3*$^{-/-}$ BMDMs were pretreated and transfected for 1 h as shown in (**b**), crude mitochondria extracts were prepared from the BMDMs and then subjected to SDD–AGE and immunoblotted with rabbit anti-MAVS antibody. **d,** WT, *Nlrp3*$^{-/-}$ or *Gpr43*$^{-/-}$

BMDMs were pretreated with or without 20 μM 4-CMTB for 24 h, crude mitochondria extracts and cell lysates were subjected to immunoblot analysis with indicated antibodies. Results represent n=2 independent experiments (**a-d**). Source data are provided as a Source Data file.

**Supplementary Table 1. Oligonucleotides Used in This Paper, Related to Methods.**

| **Name** | **Sequence (5'→3')** |
|---|---|
| **qPCR primers** | |
| *Ifna1*-F | TCTGATGCAGCAGGTGGG |
| *Ifna1*-R | AGGGCTCTCCAGACTTCTGCTCTG |
| *Ifnb1*-F | AGCTCCAAGAAAGGACGAACA |
| *Ifnb1*-R | GCCCTGTAGGTGAGGTTGAT |
| *Isg15*-F | AGCAATGGCCTGGGACCTAAA |
| *Isg15*-R | AGCCGGCACACCAATCTT |
| *Oas1a*-F | TGTCCTGGGTCATGTTAATAC |
| *Oas1a*-R | CCGTGAAGCAGGTAGAGA |
| PR8 *M1*-F | AAGACCAATCCTGTCACCTCTGA |
| PR8 *M1*-R | CAAAGCGTCTACGCTGCAGTCC |
| *Gapdh*-F | AGGTCGGTGTGAACGGATTTG |
| *Gapdh*-R | TGTAGACCATGTAGTTGAGGTCA |
| *Nlrp3*-F | AGACTGACGTCTCCGCTTTC |
| *Nlrp3*-R | CTGGTCCTTTCCTCACGGTC |
| *Gpr43-F* | GGCTTCTACAGCAGCATCTA |
| *Gpr43-R* | AAGCACACCAGGAAATTAAG |
| | ATTACCGCGGCTGCTGG |

| | |
|---|---|
| **Primers for amplifying 16S rRNA gene V3** | CGCCCGCCGCGCGCGGCGGGCGGGGCG-GGGCACGGGGGGCCTACGGGAGGCAGCAG |
| **Adaptor primers for amplifying 16S rRNA gene V3-V4** | TCGTCGGCAGCGTCAGATGTGTATAAGAG-ACAGCCTACGGGNGGCWGCAG |
| | GTCTCGTGGGCTCGGAGATGTGTATAAGA-GACAGGACTACHVGGGTATCTAATCC |
| **siRNA oligos** | |
| *Gpr43*-siRNA-F | GGACAGGGGUGGAAGUCAATT |
| *Gpr43*-siRNA-R | UUGACUUCCACCCCUGUCCTT |
| *Luciferase GL2*-siRNA-F | CGUACGCGGAAUACUUCGATT |
| *Luciferase GL2*-siRNA-R | UCGAAGUAUUCCGCGUACGTT |

**Supplementary Table 2. Regents Used in This Paper, Related to Methods.**

| REAGENT or RESOURCE | SOURCE | IDENTIFIER |
|---|---|---|
| **Chemicals** | | |
| Lipofectamine 2000 Reagent | invitrogen | Cat# 11668-019 |
| polyI:C | InvivoGen | Cat# tlrl-picw |
| True Blue™ Peroxidase Substrate (KPL) | SeraCare | Cat# 5510-0053 |
| Sodium acetate | SIGMA | Cat# S5636 |
| BSA | Shanghai yuanye Bio-Technology | Cat# S12014 |
| Red Blood Cell Lysis Buffer | Beyotime | Cat# C3702-120ml |
| β-Me | SIGMA | Cat# M3148 |
| FBS | AusGeneX | Cat# SA500S |
| Penicillin-Streptomycin | Gibco | Cat# 15140163 |
| Avertin (2,2,2-tribromoethanol) | SIGMA | Cat# T48402 |
| TRIzol Reagent® | SIGMA | Cat# T9424 |
| 4% paraformaldehyde (PFA) | Beyotime | Cat# P0099 |
| TPCK-Treated Trypsin | SIGMA | Cat# T1426 |
| $Na_3VO_4$ (Sodium orthovanadate) | SIGMA | Cat# S6508 |
| Clodronate | LIPOSOMA | Batch No. C28J0620 |
| Colloidal Microcrystalline Cellulose (Avicel®) | FMC Biopolymer | Cat# CL-611 NF |
| NaF | Sinopharm Chemical Reagent Co.,Ltd | Cat# 10019618 |
| Tween-20 | BBI Life Sciences | Cat# A600560-0500 |
| 7AAD | BD Pharmingen dilution 1:500 | Cat# 559925 |
| Protease Inhibitor Cocktail Tablets | Roche | Cat# 4693132001 |
| **Critical Commercial Assays** | | |
| Mitochondria Isolation Kit | Thermo Scientific | Cat# 89874 |

| | | |
|---|---|---|
| Mouse IFN-β ELISA kit | PBL Assay Science | Cat# 42400-1 |
| Mouse IFN-α ELISA kit | Invitrogen | Cat# BMS6027 |
| GoScript™ Reverse Transcription kit | Promega | Cat# A5001 |
| Nucleofector™ 2b Device | Lonza | Cat# AAB-1001 |
| Ingenio® 0.2 cm Cuvettes | Mirus | Cat# MIR 50121 |
| Protein A/G Magnetic Beads for IP | bimake | Cat# B23202 |
| Ingenio® Electroporation Solution | Mirus | Cat# MIR 50118 |
| **Antibodies** | | |
| Rabbit anti-IRF3 (clone D83B9) | Cell Signaling Technology dilution 1:1000 | Cat# 4302 |
| Rabbit anti-pTBK1 (Ser172, clone D52C2) | Cell Signaling Technology dilution 1:1000 | Cat# 5483 |
| Rabbit anti-TBK1 (clone D1B4) | Cell Signaling Technology dilution 1:1000 | Cat# 3504 |
| Rabbit anti-MAVS | Abcam dilution 1:1000 | Cat# ab189109 |
| Rabbit anti-β-Actin | Cell Signaling Technology dilution 1:1000 | Cat# 4967 |
| Rabbit anti-GPR43 | Bioss dilution 1:1000 | Cat# bs-13536R |
| Rabbit anti-MAVS | Cell Signaling Technology dilution 1:1000 | Cat# 4983 |
| Rabbit anti-pIRF7 (Ser477) | Invitrogen dilution 1:1000 | Cat# PA5-64834 |
| Rabbit anti-IRF7 | Invitrogen dilution 1:1000 | Cat# PA5-20280 |
| Mouse anti-NLRP3 (clone Cryo-2) | AdipoGen dilution 1:1000 | Cat# AG-20B-0014 |
| Rabbit anti-GAPDH (clone 14C10) | Cell Signaling Technology dilution 1:1000 | Cat# 2118 |

| | | |
|---|---|---|
| Mouse anti-COX IV (clone 4D11-B3-E8) | Cell Signaling Technology dilution 1:1000 | Cat# 11967 |
| Rabbit anti-β-Tubulin (clone 9F3) | Cell Signaling Technology dilution 1:1000 | Cat# 2128 |
| Anti-Mouse CD16/CD32 (clone 2.4G2) | BD Pharmingen dilution 1:100 | Cat# 553142 |
| AF488 anti-GPR43 | Bioss dilution 1:160 | Cat# bs-13536R-A488 |
| BV605 anti-Mouse CD45.2 (clone 104) | BD Horizon dilution 1:160 | Cat# 563051 |
| APC-Cy7 anti-Mouse CD11b (clone M1/70) | BD Pharmingen dilution 1:160 | Cat# 561039 |
| PE-Cy7 anti-Mouse F4/80 (clone BM8) | Biolegend dilution 1:160 | Cat# 123114 |
| Mouse anti V5-Tag (clone AMC0506) | ABcolonal dilution 1:200 | Cat# AE017 |
| Mouse anti-FLAG (clone M2) | SIGMA dilution 1:1000 | Cat# F3165 |
| Mouse anti-GFP (clone 5G4) | Cell Signaling Technology dilution 1:50 | Cat# 55494 |
| Rabbit anti-influenza A Nucleoprotein/NP | SinoBiological dilution 1:2000 | Cat# 11675-T62 |
