# Supplementary material for: Microbiota-derived acetate enhances host antiviral response via NLRP3
Source: Nat Commun. 2023 Feb 6;14:642. doi: 10.1038/s41467-023-36323-4 (PMC9901394; doi:10.1038/s41467-023-36323-4)
Supplement: Supplementary file 3 — Description of Additional Supplementary Files [file 41467_2023_36323_MOESM3_ESM.pdf]

Description of Additional Supplementary Files

File Name: Supplementary Data 1
Description: One-dimensional ^{1}H NMR spectra and changed metabolites of feces.
