# Supplementary material for: Microbiota-derived acetate enhances host antiviral response via NLRP3
Source: Nat Commun. 2023 Feb 6;14:642. doi: 10.1038/s41467-023-36323-4 (PMC9901394; doi:10.1038/s41467-023-36323-4)
Supplement: Supplementary file 5 — Reporting Summary [file 41467_2023_36323_MOESM5_ESM.pdf]

nature portfolio

Corresponding author(s): Guangxun Meng
Liping Zhao
Chenhong Zhang

Last updated by author(s): Jan 4, 2023

# Reporting Summary

Nature Portfolio wishes to improve the reproducibility of the work that we publish. This form provides structure for consistency and transparency in reporting. For further information on Nature Portfolio policies, see our Editorial Policies and the Editorial Policy Checklist.

## Statistics

For all statistical analyses, confirm that the following items are present in the figure legend, table legend, main text, or Methods section.

| n/a | Confirmed | |
|---|---|---|
| ☐ | ☒ | The exact sample size (*n*) for each experimental group/condition, given as a discrete number and unit of measurement |
| ☐ | ☒ | A statement on whether measurements were taken from distinct samples or whether the same sample was measured repeatedly |
| ☐ | ☒ | The statistical test(s) used AND whether they are one- or two-sided<br>*Only common tests should be described solely by name; describe more complex techniques in the Methods section.* |
| ☒ | ☐ | A description of all covariates tested |
| ☐ | ☒ | A description of any assumptions or corrections, such as tests of normality and adjustment for multiple comparisons |
| ☐ | ☒ | A full description of the statistical parameters including central tendency (e.g. means) or other basic estimates (e.g. regression coefficient) AND variation (e.g. standard deviation) or associated estimates of uncertainty (e.g. confidence intervals) |
| ☐ | ☒ | For null hypothesis testing, the test statistic (e.g. *F*, *t*, *r*) with confidence intervals, effect sizes, degrees of freedom and *P* value noted<br>*Give P values as exact values whenever suitable.* |
| ☒ | ☐ | For Bayesian analysis, information on the choice of priors and Markov chain Monte Carlo settings |
| ☐ | ☒ | For hierarchical and complex designs, identification of the appropriate level for tests and full reporting of outcomes |
| ☒ | ☐ | Estimates of effect sizes (e.g. Cohen's *d*, Pearson's *r*), indicating how they were calculated |

*Our web collection on statistics for biologists contains articles on many of the points above.*

## Software and code

Policy information about availability of computer code

| | |
|---|---|
| Data collection | Illumina MiSeq platform (Illumina, Inc., San Diego, CA, USA) for 16s sequencing; Bruker AVIII 600 MHz spectrometer for detecting fecal metabolites; Agilent 6890 GC ChemStation for short-chain fatty acid profiling of Bifidopseudolongum.NjM1 supernatant; ABI 7900 HT Fast Real-Time cycler (Applied Biosystems) for realtime-PCR; BD FACSDivaTM software v6 was used to collect all flow cytometry data; Olympus FV1200 was used to collect the fluorescence images. |
| Data analysis | GraphPad Prism v8.0 and IBM SPSS Statistics 23 for statistical analysis; FlowJo_v10 for flow cytometry data analysis; QIIME2 v2018.11 for 16s sequencing data analysis; LDA Effect Size (LEfSe) v1.0 identify the amplicon sequence variants (ASVs) that were significantly altered between two groups.; HGAP 2.3.0 pipeline was used to assemble subreads of the sequence into a single complete chromosome; Prokka 1.12 pipeline was used to predict and annotate protein-coding sequences (CDSs), tRNAs, and rRNAs; ImageJ v2.0 for image analysis. |

For manuscripts utilizing custom algorithms or software that are central to the research but not yet described in published literature, software must be made available to editors and reviewers. We strongly encourage code deposition in a community repository (e.g. GitHub). See the Nature Portfolio guidelines for submitting code & software for further information.

## Data

Policy information about availability of data

All manuscripts must include a data availability statement. This statement should provide the following information, where applicable:

- Accession codes, unique identifiers, or web links for publicly available datasets
- A description of any restrictions on data availability
- For clinical datasets or third party data, please ensure that the statement adheres to our policy

The authors declare that the data supporting the findings of this study are available within the article, its supplementary information files or Source Data file. The 16S rRNA gene sequences of Bifidobacterium strain NjM1 has been deposited in the Nucelotide database under accession code MW736893 (https://www.ncbi.nlm.nih.gov/nuccore/MW736893.1/?report=fasta), MW736894 (https://www.ncbi.nlm.nih.gov/nuccore/MW736894.1/?report=fasta), MW736895 (https://www.ncbi.nlm.nih.gov/nuccore/MW736895.1/?report=fasta) and MW736897 (https://www.ncbi.nlm.nih.gov/nuccore/MW736897.1/?report=fasta). The whole-genome sequence of Bifidobacterium strain NjM1 has been deposited in the Genome database under accession code PRJNA714197 (https://www.ncbi.nlm.nih.gov/nuccore/CP071805.1?report=fasta). The raw Illumine sequence data generated in this study have been deposited in the sequence read archive (SRA) database under accession code SRP310513 (https://www.ncbi.nlm.nih.gov/sra/?term=SRP310513). Source data are provided with this paper.

## Human research participants

Policy information about studies involving human research participants and Sex and Gender in Research.

| | |
|---|---|
| Reporting on sex and gender | not applicable |
| Population characteristics | not applicable |
| Recruitment | not applicable |
| Ethics oversight | not applicable |

Note that full information on the approval of the study protocol must also be provided in the manuscript.

# Field-specific reporting

Please select the one below that is the best fit for your research. If you are not sure, read the appropriate sections before making your selection.

☒ Life sciences ☐ Behavioural & social sciences ☐ Ecological, evolutionary & environmental sciences

For a reference copy of the document with all sections, see nature.com/documents/nr-reporting-summary-flat.pdf

# Life sciences study design

All studies must disclose on these points even when the disclosure is negative.

| | |
|---|---|
| Sample size | No statistical methods were used to predetermine sample size. Sample sizes were estimated based on standards of the filed and preliminary experiments. For in vitro experiment, 2 - 5 sample size was used for analysis; For in vivo experiment, 4-18 mice per group were used. These sample sizes were sufficient to detect meaningful biological difference with good reproducibility. |
| Data exclusions | No data was intentionally excluded from the analyses. |
| Replication | All experiments described were successfully replicated at least three independent times unless otherwise specified. Similar data were obtained in independent experiments. |
| Randomization | For the animal experiments, mice were randomly allocated to different experimental groups. For the cellular experiments, the same number of cells were plated for each treatment condition. |
| Blinding | For histological analysis, slides were blinded prior to scoring. The investigators were blinded to group allocation during data collection and/or analysis. |

# Reporting for specific materials, systems and methods

We require information from authors about some types of materials, experimental systems and methods used in many studies. Here, indicate whether each material, system or method listed is relevant to your study. If you are not sure if a list item applies to your research, read the appropriate section before selecting a response.

## Materials & experimental systems

| n/a | Involved in the study |
|---|---|
| | ☒ Antibodies |
| | ☒ Eukaryotic cell lines |
| ☒ | ☐ Palaeontology and archaeology |
| | ☒ Animals and other organisms |
| ☒ | ☐ Clinical data |
| ☒ | ☐ Dual use research of concern |

## Methods

| n/a | Involved in the study |
|---|---|
| ☒ | ☐ ChIP-seq |
| | ☒ Flow cytometry |
| ☒ | ☐ MRI-based neuroimaging |

# Antibodies

Antibodies used

Rabbit anti-pIRF3 (Ser396, clone 4D4G, CST, Cat# 4947, dilution 1:1000),
Rabbit anti-IRF3 (clone D83B9, CST, Cat# 4302, dilution 1:1000),
Rabbit anti-pTBK1 (Ser172, clone D52C2, CST, Cat# 5483, dilution 1:1000),
Rabbit anti-TBK1 (clone D1B4, CST, Cat# 3504, dilution 1:1000),
Rabbit anti-MAVS (Abcam, Cat# ab189109, dilution 1:1000),
Rabbit anti-β-Actin (CST, Cat# 4967, dilution 1:1000),
Rabbit anti-GPR43 (Bioss, Cat# bs-13536R, dilution 1:1000),
Rabbit anti-MAVS (CST, Cat# 4983, dilution 1:1000),
Rabbit anti-pIRF7 (Ser477, Invitrogen, Cat# PA5-64834, dilution 1:1000),
Rabbit anti-IRF7 (Invitrogen, Cat# PA5-20280, dilution 1:1000),
Mouse anti-NLRP3 (clone Cryo-2, AdipoGen, Cat# AG-20B-0014, dilution 1:1000),
Rabbit anti-GAPDH (clone 14C10, CST, Cat# 2118, dilution 1:1000),
Mouse anti-COX IV (clone 4D11-B3-E8, CST, Cat# 11967, dilution 1:1000),
Rabbit anti-β-Tubulin (clone 9F3, CST, Cat# 2128, dilution 1:1000),
Anti-Mouse CD16/CD32 (clone 2.4G2, BD Pharmingen, Cat# 553142, dilution 1:100),
AF488 anti-GPR43 (Bioss, Cat# bs-13536R-A488, dilution 1:160),
BV605 anti-Mouse CD45.2 (clone 104, BD Horizon, Cat# 563051, dilution 1:160),
APC-Cy7 anti-Mouse CD11b (clone M1/70, BD Pharmingen, Cat# 561039, dilution 1:160),
PE-Cy7 anti-Mouse F4/80 (clone BM8, Biolegend, Cat# 123114, dilution 1:160),
7AAD (BD Pharmingen, Cat# 559925, dilution 1:500),
Mouse anti V5-Tag (clone AMC0506, ABcolonal, Cat# AE017, dilution 1:200),
Mouse anti-FLAG (clone M2, SIGMA, Cat# F3165, dilution 1:1000),
Mouse anti-GFP (clone 5G4, CST, Cat# 55494, dilution 1:50),
Rabbit anti-influenza A Nucleoprotein / NP Antibody (SinoBiological, Cat# 11675-T62, dilution 1:2000).

Validation

Rabbit anti-pIRF3 (Ser396, clone 4D4G, CST, Cat# 4947, dilution 1:1000), Cell Signaling Technology; RRID: AB_823547
https://www.cellsignal.cn/products/primary-antibodies/phospho-irf-3-ser396-4d4g-rabbit-mab/4947?site-search-type=Products&N=4294956287&Ntt=4947&fromPage=plp&_requestid=1487817
Rabbit anti-IRF3 (clone D83B9, CST, Cat# 4302, dilution 1:1000), Cell Signaling Technology; RRID: AB_1904036
https://www.cellsignal.cn/products/primary-antibodies/irf-3-d83b9-rabbit-mab/4302?site-search-type=Products&N=4294956287&Ntt=4302&fromPage=plp&_requestid=1488114
Rabbit anti-pTBK1 (Ser172, clone D52C2, CST, Cat# 5483, dilution 1:1000), Cell Signaling Technology; RRID: AB_10693472
https://www.cellsignal.cn/products/primary-antibodies/phospho-tbk1-nak-ser172-d52c2-xp-rabbit-mab/5483?site-search-type=Products&N=4294956287&Ntt=5483&fromPage=plp&_requestid=1488290
Rabbit anti-TBK1 (clone D1B4, CST, Cat# 3504, dilution 1:1000), Cell Signaling Technology; RRID: AB_2255663
https://www.cellsignal.cn/products/primary-antibodies/tbk1-nak-d1b4-rabbit-mab/3504?site-search-type=Products&N=4294956287&Ntt=3504&fromPage=plp&_requestid=1488399
Rabbit anti-MAVS (Abcam, Cat# ab189109, dilution 1:1000), Cell Signaling Technology;
https://www.abcam.cn/mavs-antibody-ab189109.html
Rabbit anti-β-Actin (CST, Cat# 4967, dilution 1:1000), Cell Signaling Technology;
https://www.cellsignal.cn/products/primary-antibodies/b-actin-antibody/4967?site-search-type=Products&N=4294956287&Ntt=4967&fromPage=plp&_requestid=1488687
Rabbit anti-GPR43 (Bioss, Cat# bs-13536R, dilution 1:1000), Bioss;
https://www.biossusa.com/products/bs-13536r
Rabbit anti-MAVS (CST, Cat# 4983, dilution 1:1000), Cell Signaling Technology; RRID: AB_823566
https://www.cellsignal.cn/products/primary-antibodies/mavs-antibody-rodent-specific/4983?site-search-type=Products&N=4294956287&Ntt=4983&fromPage=plp&_requestid=1490144
Rabbit anti-pIRF7 (Ser477, Invitrogen, Cat# PA5-64834, dilution 1:1000), Invitrogen; https://www.thermofisher.cn/cn/zh/antibody/product/Phospho-IRF7-Ser477-Antibody-Polyclonal/PA5-64834
Rabbit anti-IRF7 (Invitrogen, Cat# PA5-20280, dilution 1:1000), Invitrogen;
https://www.thermofisher.cn/cn/zh/antibody/product/IRF7-Antibody-Polyclonal/PA5-20280
Mouse anti-NLRP3 (clone Cryo-2, AdipoGen, Cat# AG-20B-0014, dilution 1:1000), AdipoGen;
https://adipogen.com/ag-20b-0014-anti-nlrp3-nalp3-mab-cryo-2.html
Rabbit anti-GAPDH (clone 14C10, CST, Cat# 2118, dilution 1:1000), Cell Signaling Technology;
https://www.cellsignal.cn/products/primary-antibodies/gapdh-14c10-rabbit-mab/2118?site-search-type=Products&N=4294956287&Ntt=2118&fromPage=plp&_requestid=7217926
Mouse anti-COX IV (clone 4D11-B3-E8, CST, Cat# 11967, dilution 1:1000), Cell Signaling Technology;
https://www.cellsignal.cn/products/primary-antibodies/cox-iv-4d11-b3-e8-mouse-mab/11967?site-search-type=Products&N=4294956287&Ntt=cox+iv&fromPage=plp

Rabbit anti-β-Tubulin (clone 9F3, CST, Cat# 2128, dilution 1:1000), Cell Signaling Technology;
https://www.cellsignal.cn/products/primary-antibodies/b-tubulin-9f3-rabbit-mab/2128?site-search-type=Products&N=4294956287&Ntt=2128&fromPage=plp&_requestid=7218151
Anti-Mouse CD16/CD32 (clone 2.4G2, BD Pharmingen, Cat# 553142, dilution 1:100), BD Pharmingen;
https://www.bdbiosciences.com/zh-cn/products/reagents/flow-cytometry-reagents/research-reagents/single-color-antibodies-ruo/purified-rat-anti-mouse-cd16-cd32-mouse-bd-fc-block.553142
AF488 anti-GPR43 (Bioss, Cat# bs-13536R-A488, dilution 1:160), Bioss;
https://www.biossusa.com/products/bs-13536r-a488
BV605 anti-Mouse CD45.2 (clone 104, BD Horizon, Cat# 563051, dilution 1:160), BD Horizon;
https://www.bdbiosciences.com/en-us/products/reagents/flow-cytometry-reagents/research-reagents/single-color-antibodies-ruo/bv605-mouse-anti-mouse-cd45-2.563051
APC-Cy7 anti-Mouse CD11b (clone M1/70, BD Pharmingen, Cat# 561039, dilution 1:160), BD Pharmingen; https://www.bdbiosciences.com/en-us/products/reagents/flow-cytometry-reagents/research-reagents/single-color-antibodies-ruo/apc-cy-7-rat-anti-cd11b.561039
PE-Cy7 anti-Mouse F4/80 (clone BM8, Biolegend, Cat# 123114, dilution 1:160), Biolegend;
https://www.biolegend.com/en-us/products/pe-cyanine7-anti-mouse-f4-80-antibody-4070
7AAD (BD Pharmingen, Cat# 559925, dilution 1:500), BD Pharmingen;
https://www.bdbiosciences.com/zh-cn/products/reagents/flow-cytometry-reagents/research-reagents/single-color-antibodies-ruo/7-aad.559925
Mouse anti V5-Tag (clone AMC0506, ABcolonal, Cat# AE017, dilution 1:200), ABcolonal; RRID: AB_2770413
https://abclonal.com.cn/catalog/AE017
Mouse anti-FLAG (clone M2, SIGMA, Cat# F3165, dilution 1:1000), SIGMA;
https://www.sigmaaldrich.cn/CN/zh/product/sigma/f3165
Mouse anti-GFP (clone 5G4, CST, Cat# 55494, dilution 1:50), Cell Signaling Technology;
https://www.cellsignal.cn/products/primary-antibodies/gfp-5g4-mouse-mab/55494?site-search-type=Products&N=4294956287&Ntt=55494&fromPage=plp&_requestid=7218848
Rabbit anti-influenza A Nucleoprotein / NP Antibody (SinoBiological, Cat# 11675-T62, dilution 1:2000), SinoBiological;
https://www.sinobiological.com/antibodies/nucleocapsid-np-11675-t62

# Eukaryotic cell lines

Policy information about cell lines and Sex and Gender in Research

| | |
|---|---|
| Cell line source(s) | Human HEK293T cells ATCC ATCC CRL-3216; RRID: CVCL_0063<br>Mouse fibroblasts L929 ATCC ATCC CCL-1; RRID: CVCL_0462<br>MDCK cell line ATCC ATCC CCL-34 |
| Authentication | All cell lines were not authenticated. |
| Mycoplasma contamination | All cell lines tested negative for mycoplasma contamination. |
| Commonly misidentified lines<br>(See ICLAC register) | No commonly misidentified cell lines were used in the study. |

# Animals and other research organisms

Policy information about studies involving animals; ARRIVE guidelines recommended for reporting animal research, and Sex and Gender in Research

| | |
|---|---|
| Laboratory animals | All mice used in this study were female mice on C57BL/6 background (6-9 weeks old),. WT (wild type), Ifnar1-/- and Mavs-/- mice were originally from the Jax lab, Irf3-/- mice were originally from Dr. T. Taniguchi and shared through Dr. F. Shao37, Gpr43-/- mice were purchased from Cyagen Biotechnology Co., Ltd (https://www.cyagen.com/cn/zh-cn/sperm-bank-live/233079) and Nlrp3-/- mice had been described before38,39. The mice were routinely maintained in a pathogen-free animal facility at a temperature of 21℃, relative humidity of 50%-70%, and under a constant 12 h light/dark cycle, and were given free access to food and water throughout the study at Institut Pasteur of Shanghai. Cohousing (Co-) of WT and Nlrp3-/- mice were carried out at 1:1 ratio since weaning (3 weeks old) till adulthood (9 weeks old), or the mice were separated upon weaning according to genotype, denoted as singly housed (Single-). |
| Wild animals | This study did not involve wild animals. |
| Reporting on sex | All animal experiments were conducted on female mice. |
| Field-collected samples | The study did not involve samples collected from the field. |
| Ethics oversight | All animal experiments were performed in compliance with the Regulations for the Care and Use of Laboratory Animals issued by the Ministry of Science and Technology of the People's Republic of China, which enforces the ethical use of animals. The protocol was approved by Institutional Animal Care and Use Committee (IACUC) at Institut Pasteur of Shanghai, Chinese Academy of Sciences (Permit Number: P2019014). |

Note that full information on the approval of the study protocol must also be provided in the manuscript.

# Flow Cytometry

## Plots

Confirm that:

☒ The axis labels state the marker and fluorochrome used (e.g. CD4-FITC).

☒ The axis scales are clearly visible. Include numbers along axes only for bottom left plot of group (a 'group' is an analysis of identical markers).

☒ All plots are contour plots with outliers or pseudocolor plots.

☒ A numerical value for number of cells or percentage (with statistics) is provided.

## Methodology

| | |
|---|---|
| Sample preparation | Cells from BALF or BMDMs were washed with FACS buffer (1×PBS containing 0.5% BSA and 2 mM EDTA) and then blocked with FcR blocking antibody (anti-Mouse CD16/CD32 (clone 2.4G2, BD Pharmingen, Cat# 553142, dilution 1:100) before surface staining with BV605 anti-Mouse CD45.2 (clone 104, BD Horizon, Cat# 563051, dilution 1:160), APC-Cy7 anti-Mouse CD11b (clone M1/70, BD Pharmingen, Cat# 561039, dilution 1:160), PE-Cy7 anti-Mouse F4/80 (clone BM8, Biolegend, Cat# 123114, dilution 1:160) and AF488 anti-GPR43 (Bioss, Cat# bs-13536R-A488, dilution 1:160). For intracellular Ca2+ detection, BMDMs were treated with 250 µM sodium acetate (SA) (S5636, Sigma) for 24 h, washed with PBS three times, and then incubated with 2 µM of Fluo-4 AM (S1060, Beyotime) for 45 min at 24℃. Dead cells were excluded using 7AAD (BD Pharmingen, Cat# 559925, dilution 1:500) and data were acquired using a BD Fortessa flow cytometer with BD FACSDivaTM software v6 and then analyzed with FlowJo_v10 software. |
| Instrument | Samples were acquired on a Fortessa (BD). |
| Software | BD FACSDiva for data acquisition, and data were analyzed using FlowJo v10.6.2 (Flowjo. LLC). |
| Cell population abundance | No FACS sorting was performed in this study. |
| Gating strategy | Cell debris were excluded by SSC-A and FSC-A gating (Cells), doublets were excluded by FSC-A and FSC-H gating (Singlets), dead cells were excluded by FSC-A and 7AAD gating (Live cells), live cells were gated on leukocyte population (CD45+), and the subset of macrophages was estimated according to the markers CD11b and F4/80. Mean fluorescence intensity of GPR43 on CD11b+F4/80+ macrophages was calculated by adding statistic Mean of Comp-AF488-A. The gating strategy is also described in the figure legends (Supplementary Fig. 6a). |

☒ Tick this box to confirm that a figure exemplifying the gating strategy is provided in the Supplementary Information.
